# Therapeutic Uses of Gaming in Mental Health: An Untapped Potential

**DOI:** 10.2196/57714

**Published:** 2024-04-25

**Authors:** Jens Peter Eckardt

**Affiliations:** 1 Bedre Psykiatri Research Unit (Videnscenter) Copenhagen Denmark

**Keywords:** digital mental health interventions, mental health, psychiatry, gaming, serious games, casual video games, commercial games, exergames, adolescent, anxiety, teenage, video game, youth

In their exploratory study, Pine et al [1] unveil promising results indicating the potential therapeutic benefits of using casual video games. While caution is necessary, particularly concerning the interpretation of student feedback, self-assessment effectiveness, recruitment, pandemic effects, and the absence of mental distress screening, the study demonstrates that video games integrating brief mental health messages provide a promising approach to merging digital intervention with the accessibility of commercial gaming. Although a randomized controlled trial is also required for precise clinical impact assessment, these preliminary findings bolster the notion that “gaming” (primarily commercial video games, serious games, and exergames) within mental health services is validated as a viable alternative or complement to conventional methods of prevention, assessment, and treatment.

Gaming, in a broad sense, is one of the most popular leisure activities globally, estimated to involve millions of gamers worldwide [2], making it ubiquitous and omnipresent, regardless of whether one has a mental illness or not. Considering the growing disparity between demand and supply for mental health assistance, combined with factors such as high disease burden, treatment costs, and long waiting lists, new alternative solutions must be explored. Coupled with accelerating technology-based game development and popularity, it may just be a matter of time before gaming truly disrupts several aspects of psychiatric work.

At present, gaming research has been conducted in the context of different psychiatric disorders such as anxiety, depression, eating disorders, attention-deficit/hyperactivity disorder (ADHD), stress symptoms, posttraumatic stress disorder (PTSD), autism, phobias, and schizophrenia, as well as in forensic psychiatry. The results vary, but reduced symptomatology, improved social, executive, and cognitive functions, as well as improved attention processes and problem-solving, have been reported. Gaming has also proven effective in offering temporary distraction from serious events, and it fosters social communities [3].

However, there are several challenges to research and practical application of gaming in mental health services. Moreover, there are critical concerns regarding the limited number of high-quality studies; weak research designs; methodological issues; and questions about generalizability, causality, mechanisms of action, control groups, effect sizes, definitions, terminology, comparability, theoretical strength, harmful effects, and transferability [2,3]. In addition, critics highlight concerns, such as gaming disorders as outlined in the *ICD-11 (International Classification of Diseases 11th Revision)*, prolonged sedentary screen time, exposure to violence, and instances of excessive or problematic gaming behavior [2,4]. Furthermore, critics argue that gaming encourages avoidance tactics, hindering physical interactions within communities. This challenge is compounded by distant communication, escapism, isolation, loneliness, emotional detachment, addiction, sleep disturbances, and physical inactivity, all posing risks of worsening the individual’s condition. Critics argue gaming is not a treatment strategy but rather a tool for enhancing communication and presence among individuals.

Research and applications of gaming in psychiatry are expanding and proving beneficial for specific patient demographics, yet there is a pressing need for a more robust knowledge base to fully grasp both the potentials and challenges involved [5]. Capitalizing on these opportunities for clinical use will demand innovative thinking within multidisciplinary research environments [2]. In conclusion, it is evident that gaming, which is deeply embedded in our culture, possesses promising yet unexplored avenues to emerge as a vital component in forthcoming treatments for mental disorders.

## Abbreviations

ADHD: attention-deficit/hyperactivity disorder
*ICD-11*: *International Classification of Diseases 11th Revision*
PTSD: posttraumatic stress disorder
